# Anti-Inflammatory Effects of Phenolic Compounds Isolated from *Quercus Mongolica* Fisch. ex Ledeb. on UVB-Irradiated Human Skin Cells

**DOI:** 10.3390/molecules24173094

**Published:** 2019-08-26

**Authors:** Jun Yin, Han Hyuk Kim, In Hyeok Hwang, Dong Hee Kim, Min Won Lee

**Affiliations:** 1 Department of Pharmacognosy and Natural product-derived Medicine, College of Pharmacy, Chung-Ang University, Seoul 156-756, Korea; 2 R&D Department Applied Product Development Team, Traditional Korean Medicine Technology Division, 94, Hwarang-ro(Gapje-dong), Gyeongsan-si, Gyeongsangbuk-do 38540, Korea

**Keywords:** *Quercus mongolica* Fisch. ex Ledeb., pedunculagin, skin inflammation disease

## Abstract

*Quercus mongolica* Fisch. ex Ledeb. (QM) has been used as an oriental traditional medicine to relieve hemorrhoids, fever, and enteritis. We screened the inhibitory activities of the extracts and compounds (**1**–**6**) isolated from QM on the production of inflammatory cytokines and chemokines to evaluate their anti-inflammatory activities. Further, we evaluated the expression levels of cytokines, chemokines, and immune factors on pedunculagin (PC, **1**), which was selected from isolated compounds (**1**–**6**) because of its potential anti-inflammation effect. Additionally, we evaluated whether the inflammation mitigation effects of PC (**1**) following UVB exposure in keratinocytes occurred because of nuclear factor (NF)-κB and signal transducer and activator of transcription (STAT)/Janus kinase (JAK) activation. PC (**1**) remarkably suppressed interleukin (IL)-6, IL-10, IL-13, and monocyte chemoattractant protein-1 (MCP-1) mRNA expression and reduced the mRNA expression level of Cyclooxygenase-2 (COX-2) and also reduced the phosphorylation of p38 mitogen-activated protein kinases (p38), c-Jun N-terminal kinase (JNK), and extracellular signal-regulated kinase (ERK) in a concentration-dependent manner.

## 1. Introduction

Keratinocytes exposed to ultraviolet B (UVB) cause various inflammatory skin diseases, such as sunburn, psoriasis, and skin cancer [1,2]. Keratinocytes irradiated with UVB express and release inflammatory mediators in response to skin inflammation as well as pro-inflammatory cytokines during the progression phase of the inflammatory process, such as IL-6, IL-8, IL-10, and IL-13 [3,4,5]. Keratinocytes also release thymus and activation-regulated chemokine (TARC/CCL17) and macrophage-derived chemokine (MDC/CCL22), which is closely related to TARC [6]. TARC and MDC may play important roles in the development of chronic inflammation [7]. Further, UVB and pro-inflammatory mediators induce nuclear factor (NF)-κB activation, which is an important nuclear transcription factor that induces inflammation and immune activities [8,9,10]. Moreover, some mitogen-activated protein kinase (MAPK) pathways can also induce NF-κB activation, such as p38 and extracellular signal-regulated kinase (ERK) pathways, which are known to promote cell growth, proliferation, survival, and DNA damage [8,9,11]. Thus, keratinocytes play an important role in the pathogenesis of inflammatory skin diseases, like atopic dermatitis and psoriasis [12].

*Quercus mongolica* Fisch. ex Ledeb. (QM) is a species of deciduous oak native to Korea. QM exhibits anti-allergic, anti-microbial, and anti-oxidation activities and has been used to treat hemorrhoids, fever, and enteritis as an oriental traditional medicine [13,14,15,16]. Previous studies of the chemical composition of QM led to the isolation of various triterpenoid, tannins, flavonoids, and phenol glucoside gallates exhibiting a variety of bioactivities, including anti-oxidative, anti-tumor, and anti-fungal activities [17,18,19,20]. Previously, we isolated several phenolic compounds from QM, and evaluated its anti-photoaging activity [19].

In this study, to evaluate the anti-inflammatory activities of QM, we measured the inhibition of inflammatory chemokine (TARC and MDC) and cytokine (MCP-1, IL-6, IL-8, IL-10, and IL-13) production by extracts and compounds (**1**–**6**) isolated from QM. Further, we evaluated the expression of MCP-1, MDC, TARC, IL-6, IL-8, IL-10, and IL-13 and immune factors, such as COX-2, on PC (**1**) with potential regarding anti-inflammatory activities in keratinocytes irradiated with UVB. Additionally, we examined whether the inflammation mitigation effects of PC (**1**) on UVB exposure in keratinocytes were due to NF-κB and STAT/JAK activation.

## 2. Results

### 2.1. Phytochemicals from QM

In this study, the extracts of QM and compounds (**1**–**6**)—one ellagitannin [pedunculagin (PC, **1**)], one gallotannin [(+)-gallocatechin (**2**)], one flavan-3-ol [(+)-catechin (**3**)], and three flavonoids [quercetin-3-*O*-(6″-*O*-galloyl)-*β*-d-glucopyranoside (QGG, **4**), kaempferol-3-*O*-*β*-d-glucopyranoside-7-*O*-*α*-l-rhamnopyranoside (**5**), kaempferol-3-*O*-(6″-galloyl)-*β*-d-glucopyranoside (**6**)]—isolated previously [19] were used for evaluating its anti-inflammatory activities.

### 2.2. Inhibition of Chemokine and Cytokine Production

UVB irradiation causes inflammation by inducing the activation of various chemokines and cytokines, which are immune regulators produced by human keratinocyte cells. Keratinocytes (5 × 10^6^ cells/mL) were treated with Lipopolysaccharides (LPS) for 30 min at 37 °C. Keratinocyte cytosolic and nuclear extracts were prepared as previously described [21]. To evaluate the effects of QM extracts and compounds on immune stimulation, we measured the expression levels of chemokines and cytokines. The ethyl acetate (EtOAc) layer showed the strongest activity compared to the other fractions (Table 1). Compound **1**, which was isolated from the EtOAc layer, was the most effective inhibitor of inflammatory cytokines and chemokines (Table 2).

### 2.3. mRNA Expression of Chemokines and Cytokines

**Figure 1:**
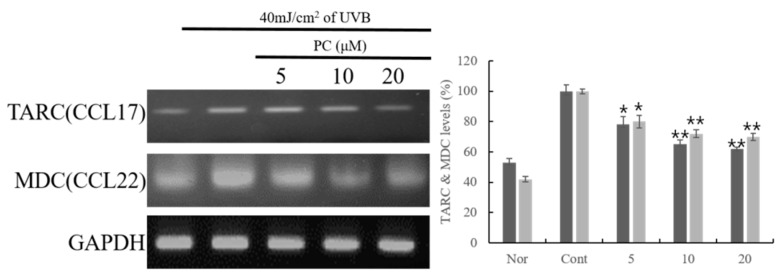
Effect of PC (**1**)-treated HaCaT cells on MDC and TARC expression. HaCaT cells were pre-treated with different concentrations of PC (**1**) (5, 10, 20 μM) for 1 h. The cells were further stimulated with 40 mJ/cm^2^ UVB. After 6 h, the cells were harvested, and relative mRNA levels were determined. Histograms show the densitometric data for TARC and MDC mRNA normalized to the level of GAPDH. Each value represents the mean ± SD of three individual experiments. Nor: Non-treated cell group (0 h), Cont: 40 mJ/cm^2^ UVB-treated cell group. Data are expressed as the means ± S.D., *n* = 3. * *p* < 0.001 and ** *p* < 0.0001 compared to the control group.

**Figure 2:**
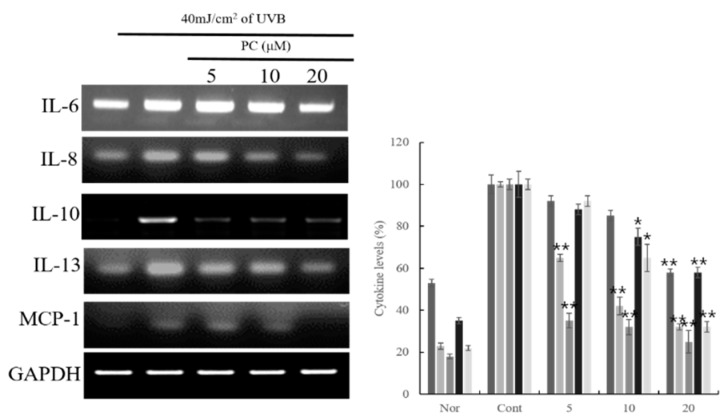
Effect of PC (**1**) treatment on cytokine mRNA expression in HaCaT cells. HaCaT cells were treated with different concentrations of PC (**1**) (5, 10, 20 μM) for 1 h. The cells were further stimulated with 40 mJ/cm^2^ UVB. After 6 h, the cells were harvested, and relative mRNA levels were determined. Histograms show the densitometric data for TARC and MDC mRNA normalized to GAPDH. Each value represents the mean ± SD of three individual experiments. Nor: Non-treated cell group (0 h), Cont: 40 mJ/cm^2^ UVB treatment cell group. Data are expressed as the means ± S.D., *n* = 3. * *p* < 0.001 and ** *p* < 0.0001 compared to the control group.

Subsequent experiments were conducted to evaluate cytokine and chemokine mRNA levels following treatment with PC (**1**), which showed the best inhibitory activities on inflammatory chemokine and cytokine among the compounds (**1**–**6**). PC (**1**) showed potent inhibitory activities on the expression of chemokines and cytokines, including TARC and MDC, and IL-6, 8, 10, 13, and MCP-1 (Figure 1 and Figure 2). In particular, PC (**1**) dose-dependently suppressed IL-6, IL-10, and IL-13 mRNA expression. Further, at a dose of 20 μM, PC (**1**) significantly inhibited IL-8, IL-10, and IL-13. At the mRNA level, PC (**1**) dose-dependently inhibited UVB-induced MCP-1 (Figure 2).

### 2.4. mRNA Expression of COX-2

**Figure 3:**
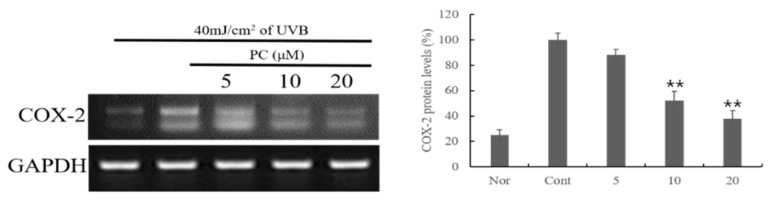
Effect of PC (**1**) treatment on COX-2 mRNA expression in HaCaT cells. HaCaT cells were treated with different concentrations of PC (**1**) for 1 h. The cells were further stimulated with 40 mJ/cm^2^ UVB. After 24 h, the cells were harvested, and relative mRNA levels were determined. Histograms show the densitometric data of COX-2 mRNA normalized to GAPDH. Each value represents the mean ± SD of three individual experiments. Nor: Non-treated cell group (0 h), Cont: 40 mJ/cm^2^ UVB-treated cell group. Data are expressed as the means ± S.D., *n* = 3. ** *p* < 0.0001 compared to the control group.

In unstimulated HaCaT cells, COX-2 mRNA expression was nearly undetectable. In contrast, UVB-irradiated HaCaT cells showed drastically increased COX-2 mRNA expression levels. The addition of exogenous PC (**1**) significantly reduced UVB-irradiated COX-2 mRNA expression (Figure 3).

### 2.5. Inhibitory Activity on Phosphorylation of p38/JNK/ERK/IκB and Signaling Pathways Activating STAT/JAK and NF-κB

**Figure 4:**
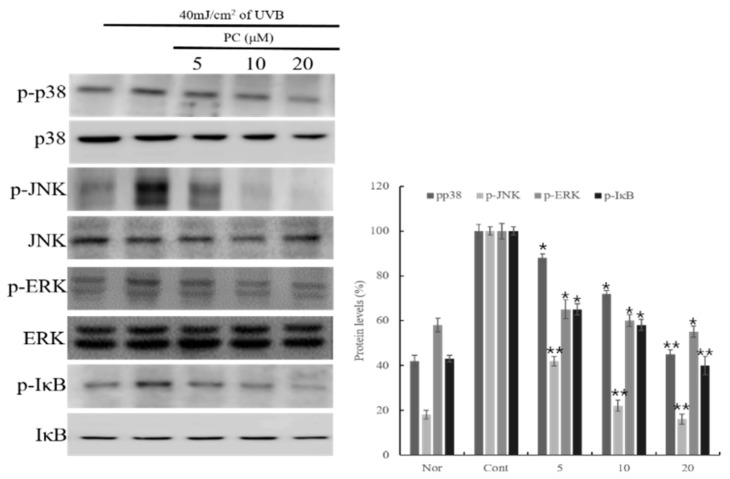
Effect of PC (1) treatment on MAPK expression in HaCaT cells. HaCaT cells were pre-treated different concentrations of PC (1) (5, 10, 20 μM) for 1 h. The cells were further stimulated with 40 mJ/cm^2^ UVB. After 6 h, the cells were harvested, and the relative protein levels were determined. Histograms show the densitometric data of p-p38, p-JNK, p-ERK, and p-IκB protein normalized to GAPDH. Each value represents the mean ± SD of three individual experiments. Nor: Non-treated cell group (0 h), Cont: 40 mJ/cm^2^ UVB-treated cell group. Data are expressed as the means ± S.D., *n* = 3. * *p* < 0.001 and ** *p* < 0.0001 compared to the control group.

We investigated whether PC (**1**) influences UVB-irradiated HaCaT cells by western blotting. Cells were stimulated with UVB for 5 to 30 min, after which the levels of p38/JNK/ERK phosphorylation were determined. The results revealed that 20 μM PC (**1**) inhibited p38, JNK, and ERK expression and the phosphorylation of IκBα (Figure 4). 

**Figure 5:**
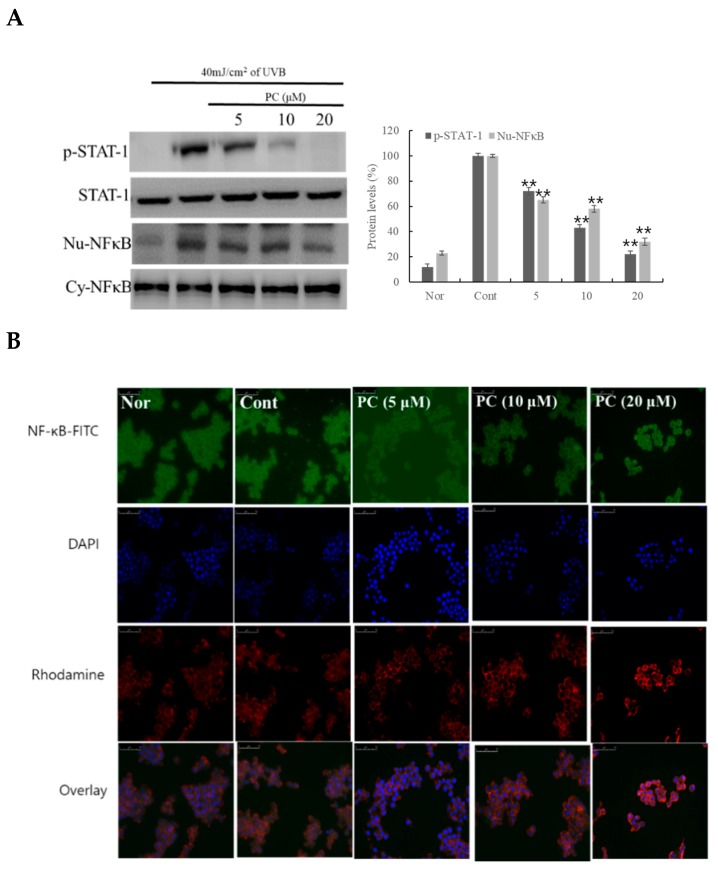
Effect of PC (1) treatment on STAT and NF-κB protein expression in HaCaT cells. HaCaT cells were pre-treated with different concentrations of PC (1) (5, 10, 20 μM) for 1 h. The cells were further stimulated with 40 mJ/cm^2^ UVB light. After 6 h, the cells were harvested, and relative protein levels were determined. Histograms show the densitometric data of STAT and NF-κB protein normalized to GAPDH. Each value represents the mean ± SD of three individual experiments. Nor: Non-treated cell group (0 h), Cont: 40 J/cm^2^ UVB-treated cell group. **A**: STAT and NF-κB protein levels, **B**: NF-κB-FITC, DAPI, Rhodamine and Overlay expression. Data are expressed as the means ± S.D., *n* = 3. ** *p* < 0.0001 compared to the control group.

Further, we investigated the effect of PC (**1**) on UVB-irradiated NF-κB translocation and STAT1 phosphorylation. PC (**1**) dose-dependently inhibited the translocation of NF-κB from the cytoplasm to the nucleus (Figure 5A). PC (**1**) showed stronger inhibitory activities than the group treated with UVB irradiation alone, a potent pharmacological inhibitor of NF-κB translocation into the nucleus. This approach has been suggested as an indirect method for controlling inflammation-related disease. Finally, PC (**1**) dose-dependently decreased the expression of p-STAT1 (Figure 5B). 

### 2.6. Cell Migration

**Figure 6:**
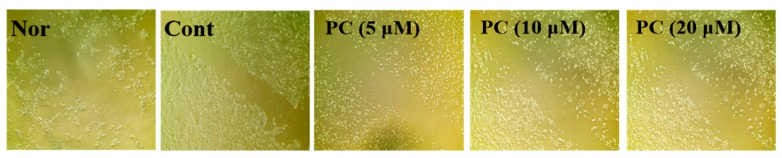
Increased cell proliferation and migration activities of PC (1)-treated HaCaT cells. HaCaT cells were scratched with a yellow tip. Migration levels of HaCaT cells were observed using an optical microscope and photographs were obtained. HaCaT cells were treated with different concentrations of PC (1) (5, 10, 20 μM) for 24 h. PC (1) treatment led to an increase in the migration of HaCaT cells. Nor: Non-treated cell group (0 h), Cont: 40 mJ/cm^2^ UVB-treated cell group.

The effects of PC (**1**) on the migration ability of HaCaT cells were evaluated in a wound healing assay. HaCaT cells uniformly grown as a monolayer were scratched, and all cells in the solid line were removed. As a result, control group cells showed some migration ability, while the group treated with PC (**1**) showed dose-dependent increases in migration capacities. Particularly, the cell migration capacity following treatment with 20 μM PC (**1**) was increased (Figure 6).

## 3. Discussion

Inflammatory diseases are manifested by a deregulated skin barrier function and a disruption of the Th2/Th1 immune balance [22,23]. Crosstalk between immune and genetic factors is involved in inflammation-related diseases, such as atopic and contact dermatitis, a particularly difficult condition to treat. Among environmental factors, UV light induces inflammatory reactions characterized by increases of many chemokines and cytokines. We therefore examined the effect of PC (**1**) on Th2 immune modulation. PC (**1**) effectively suppressed increased Th2 activation as determined from its inhibitory effect on UVB-irradiated production of TARC and MDC. TARC and MDC, members of the chemokine subfamily, are expressed by keratinocytes. These selectively control the refection and migration of Th2 lymphocytes to inflammatory sites and are considered as major factors in the pathogenesis of inflammatory disease, such as atopic dermatitis [7,24]. Other cytokines function as signaling peptides to regulate the cell cycle and control tissue-specific cell homing. In the skin, chemokines are secreted by resident cells. Chemokines and cytokines participate in the induction and maintenance of inflammation in the skin [25]. Moreover, inflammatory diseases are characterized by an enhanced production of MCP-1, a member of the CC-chemokine family. MCP-1 directs the production of leukocytes to the inflammatory focus [26]. These results demonstrate the potential use of PC (**1**) to treat skin inflammatory diseases.

UVB light induces the generation of reactive oxygen species (ROS), which play a key role in enhancing inflammation by activating the NF-κB and activator protein-1 transcription factors and increasing COX-2 expression in various inflammatory diseases [27,28]. Therefore, the effect of PC (**1**) on the expression of COX-2 mRNA was also evaluated to determine the anti-inflammatory activity of the treatments. The potent inhibitory activity of COX-2 mRNA expression on PC (**1**) strongly suggest that PC (**1**) plays an anti-inflammatory role in keratinocytes.

MAPKs are known for directing intracellular response signaling pathways to various stimuli, such as mitogens, and creating an inflammatory response to regulate mitosis, cellular differentiation, cell survival, and inflammation stress [29,30,31,32]. The stimulations could induce target gene expression by activation p38, JNK, and ERK transcription factors. p38 kinases are responsive to stimuli to regulate an inflammation response, such as IL-6 and IL-8 production and the expression of nitric oxide and metalloproteinase [33]. c-Jun N-terminal kinases (JNKs) phosphorylate c-Jun and increase activator protein-1 to regulate the inflammatory response [34]. The MAPK/ERK pathway, also known as the Ras-Raf-MEK-ERK pathway, communicates a signal from a cell surface receptor to the cell nucleus DNA, such as translocation of NF-κB to express inflammation genes [35]. In addition, it has been reported that various chronic inflammation skin diseases, such as atopic dermatitis, are induced by activation and expression of pro-inflammatory cytokines through NF-κB and JAK/STAT signaling pathways [36,37,38]. All these factors are closely associated with chemokine and cytokine production in UVB-irradiated HaCaT cells. Therefore, the anti-inflammatory effect of PC (**1**) by controlling the expression of inflammatory factors through p38, JNK, and ERK demonstrates that PC (**1**) activates molecular events that block the translocation of NF-κB and activation of STAT1.

Various cytokines and growth factors produced in keratinocytes maintain the homeostasis of the skin and are involved in immune and inflammatory reactions and cell proliferation [39]. When inflammation responses are induced by inflammatory mediators, cells were recruited and activated to the inflamed site [40]. This result supports that PC (**1**) improves anti-inflammation by increasing the cell migration ability of keratinocytes.

## 4. Materials and Methods 

### 4.1. Plant Material

The leaves of QM were collected from the Korea National Arboretum in Pocheon, Gyeong-gi, Republic of Korea in October 2011. The plant was identified by Dr. Sung-Sik Kim (Korea National Arboretum). A voucher specimen (QM 2011-10) has been deposited at the herbarium of the College of Pharmacy, Chung-Ang University.

### 4.2. Extract Preparation and Compound Identification

QM extract preparation and compound identification has been described previously [19]. The air-dried QM leaves (4.2 kg) were extracted with 80% acetone to yield QM extract (400 g), and were partitioned into n-hexane fraction (22.7 g), EtOAc fraction (35.5 g), and water fraction (240 g). The EtOAc fraction was further fractionated by column chromatography to afford five subfractions, Fr.1 (4.8 g), Fr.2 (5.4 g), Fr.3 (7.9 g), Fr.4 (8.1 g), and Fr.5 (5.3 g). Fr.1 was further chromatographed over a Sephadex LH-20 and ODS column to yield pedunculagin (**1**, 470 mg) and (−)-epigallocatechin (**2**, 105 mg). Fr.2 was chromatographed over Sephadex LH-20 and ODS to yield (+)-catechin (**3**, 59 mg) and quercetin 3-*O*-(6″-*O*-galloyl)-*β*-d-glucopyranoside (**4**, 56 mg). Similarly, Fr.3 was chromatographed over ODS to yield kaempferol-3-*O*-*β*-d-glucopyranoside-7-*O*-*α*-l-rhamnopyranoside (**5**, 98 mg). Fr.4 was chromatographed over ODS to yield kaempferol 3-*O*-(6″-galloyl)-*β*-d-glucopyranoside (**6**, 378 mg). The structures of the compounds (**1**–**6**) were identified by analysis of ^1^H and ^13^C NMR spectra and comparison with references (Figures S1–S9). These fractions, compounds, and QM extract were used to study the anti-inflammatory activity on UV-B irradiated human skin cells, presently.

### 4.3. General Experimental Procedure

Dulbecco’s modified Eagle medium (DMEM), fetal bovine serum, and streptomycin-penicillin were purchased from Gibco (Grand Island, NY, USA). Recombinant human IL-6, IL-8, IL-10, IL-13, and macrophage-derived chemokine (MDC) and TARC Quantikine ELISA kits were purchased from R&D Systems (Minneapolis, MN, USA). The nuclear and cytoplasmic extraction reagents and first-strand cDNA synthesis kit were purchased from Thermo Scientific (Waltham, MA, USA). The primary antibodies for p38, c-Jun N-terminal kinase (JNK), and ERK were purchased form Abcam (Cambridge, UK). Antibodies against NF-κB, inhibitory kappa B alpha (IκBα), phosphorylated IκBα, phosphorylated signal transducer and activator of transcription 1 (p-STAT-1), and signal transducer and activator of transcription 1 (STAT-1) were purchased from Cell Signaling Technology (Danvers, MA, USA). PVDF membrane, TEMED, SDS, and acrylamide were purchased from Bio-Rad (Hercules, CA, USA). 

### 4.4. Cell Culture

Immortalized human keratinocytes (HaCaT) were purchased from the American Type Culture collection (Manassas, VA, USA). The cells were were grown at 37 °C in a 5% CO_2_ humidified atmosphere in high-glucose DMEM containing 10% fetal bovine serum and 1% streptomycin/penicillin. 

### 4.5. Immunoassays for Cytokines and Chemokines

Keratinocytes (1 × 10^5^ cells/300 μL for the cytokine assay or 5 × 10^5^ cells/400 μL for the chemokine assay) were grown in a 24-well plate and treated with 1 μg/mL LPS and samples for 24 h. After centrifugation at 412× *g* for 10 min, IL-6, IL-8, IL-10, IL-13, TARC, and MCP-1 levels in the culture supernatant was analyzed using the ELISA kits and the absorbance was measured at 450 nm using a microplate reader (Tecan, Ltd., Salzburg, Austria).

### 4.6. Total RNA Extraction and Reverse Transcription Polymerase Chain Reaction (RT-PCR)

After treatment of the samples, total RNA was extracted using TRIzol Reagent (Molecular Research Centre, Ohio, USA). Briefly, 2 µg total RNA was reverse transcribed into cDNA using a first strand cDNA synthesis kit (Fermentas, Waltham, MA, USA). The cDNA was used as a template for PCR amplification; 28 cycles of PCR were carried out using a DNA engine gradient cycler (MJ Research, Inc., Waltham, MA, USA). This was followed by denaturation for 30 s at 95 °C, annealing at 55 to 60 °C for 1 min, and extension at 72 °C for 30 s. GAPDH was used as an internal control. The sequences of the PCR primers used are shown below (Table 3).

### 4.7. Preparation of Cytosolic and Nuclear Extracts

After treatment of the samples, keratinocytes were harvested by centrifugation at 412× *g* for 10 min and washed twice with phosphate-buffered saline (PBS). The cells were suspended in 400 μL lysis buffer (10 μM KCl, 1.5 μM MgCl_2_, 0.1 μM EDTA, 0.1 μM EGTA, 1 μM dithiothreitol, 0.5 μM PMSF, 1 μM sodium orthovanadate, 2 μg/mL aprotinin, 2 μg/mL leupeptin, and 10 mM Hepes-KOH, pH 7.8) and were allowed to swell on ice for 15 min. Next, 25 μL of a 10% Nonidet NP-40 solution (final concentration approximately 0.6%) was added, and the tubes were vigorously vortexed for 10 s. The homogenates were centrifuged at 12,000× *g* for 10 min at 4 °C. The supernatants were stored as cytoplasmic extracts at −70 °C. The nuclear pellets were resuspended in 50 μL of ice-cold hypertonic solution containing 5% glycerol and 0.4 M NaCl lysis buffer. The tubes were incubated on ice for 30 min and then centrifuged at 12,000× *g* for 15 min at 4 °C. The supernatants were collected as the nuclear extracts and stored at −70 °C. Protein concentrations were determined using the Bradford method according to the manufacturer’s instructions (Bio-Rad Laboratories, Hercules, CA, USA). 

### 4.8. Western Blot Assay

After treatment of the samples, the cells were washed with ice-cold PBS, and treated with homogenizing buffer (Roche Diagnostics, Basel, Switzerland) and protease inhibitor (Roche Diagnostics, Indianapolis, IN). Cell lysates were centrifuged at 13,475× *g* for 10 min and the supernatants were collected. Protein concentrations were determined by using Bradford protein assay reagent (Bio-Rad Laboratories); 20 µg of protein was loaded and separated on a 7.0% to 10% SDS gel and then transferred onto a PVDF membrane, which was probed with specific primary antibodies overnight, and incubated with secondary antibodies for 1 hour at room temperature. Western blots were developed by enhanced chemiluminescence (Amersham Biosciences, Buckinghamshire, UK) and quantified using a Gel-pro analyzer (Media Cybernetics, Inc., Rockville, MD, USA).

### 4.9. Immunofluorescence

Cells were aliquoted in an 8-well Lab-Tek chamber (Nalge-Nunc, enfield, NY, USA) with 1 × 10^3^ cells and grown for 24 h after samples were treated. The cells were washed with ice-cold PBS, after which 95% Triton X-100 was added and incubated for 10 min. After washing, 1% of bovine serum albumin was added and incubated for 1 hour and the NF-κB primary antibody (1:100) was added and incubated at 4 °C overnight. The secondary antibody was Alexa 488-conjugated goat anti-mouse IgG (Invitrogen, Carlsbad, CA, USA) (fluorescein isothiocyanate) (1:1000). Stained cells were mounted on slides after washing with PBS and observed by fluorescent microscopy to determine NF-κB activity.

### 4.10. Cell Migration Assay

Cell migration was measured in a scratch wound assay. HaCaT cells were seeded at a density of 5 × 10^5^ cells in a 12-well plate and cultured to >90% confluence. The cells were pre-treated for 1 h and then exposed to 40 mJ/cm^2^ of UVB radiation and scratched using a 200-μL micropipette tip. After treating samples for 24 h, images of the scratch wound were acquired using a phase contrast microscope.

### 4.11. Statistical Analysis

Analysis of variance was performed using SPSS software (SPSS, Inc., Chicago, IL, USA). All data are expressed as the mean ± SD, and statistically significant differences between experimental group and control group values were analyzed by one-way analysis of variance followed by the *t*-test. * A *p*-value < 0.001 and ** *p*-value < 0.0001 were considered statistically significant.

## 5. Conclusions

QM and the isolated compounds (**1**–**6**) showed inhibitory activities towards inflammatory cytokines and chemokines. The EtOAc fraction of QM and PC (**1**) among the isolated compounds (**1**–**6**) showed potent activities against MCP-1, TARC, IL-6, IL-8, IL-10, and IL-13 in keratinocytes irradiated with UVB.

PC (**1**) relieved inflammation by enhancing the regeneration of keratinocytes exposed to UVB. PC (**1**) also inhibited chemokine and cytokine overexpression induced by UV irradiation in keratinocytes in a concentration-dependent manner. Additionally, we examined whether the result of inflammation relaxation of PC (**1**) occurred because of the activity of NF-κB and STAT/JAK in UVB-irradiated keratinocytes. PC (**1**) not only reduced p38, JNK, and ERK phosphorylation in a concentration-dependent manner but also reduced the expression of NF-κB and STAT1, indicating that PC (**1**) inhibits the production of chronic inflammatory disease factors induced by UVB irradiation. These results suggest that **1** and QM can be further developed to treat chronic inflammatory skin diseases, like atopic dermatitis and psoriasis.

## Figures and Tables

**Table 1:** Inhibition of chemokine and cytokine production by QM extracts and fractions.

Chemokine and Cytokine	QM Extract	*n*-Hexane Layer	EtOAc Layer	H_2_O Layer
	IC_50_ (μg/mL)			
MCP-1	32.9 ± 2.7 ^b^	<100	17.0 ± 1.5 ^a^	36.3 ± 0.4 ^b^
TARC	36.0 ± 1.7 ^b^	<100	16.1 ± 0.8 ^a^	49.9 ± 0.7 ^b^
IL-6	37.1 ± 0.9 ^b^	<100	25.0 ± 1.1 ^a^	56.3 ± 2.1 ^c^
IL-8	27.4 ± 1.1 ^b^	<100	20.5 ± 1.9 ^a^	45.5 ± 1.5 ^b^
IL-10	15.8 ± 1.3 ^a^	<100	9.3 ± 2.2 ^a^	55.6 ± 2.3 ^b^
IL-13	21.1 ± 2.2 ^a^	<100	5.8 ± 0.4 ^a^	65.0 ± 1.7 ^b^

Values represent the means ± S.D. of three determinations. Values with different superscripts in the same columns are significantly different (*p* < 0.05).

**Table 2:** Inhibition of chemokine and cytokine production by compounds **1**–**6**.

Chemokine and Cytokine	1	2	3	4	5	6
		IC_50_ (μM)				
MCP-1	8.5 ± 2.3 ^a^	78.7 ± 1.1 ^d^	74.2 ± 5.5 ^b^	13.7 ± 4.1 ^ab^	<100	15.2 ± 1.1 ^ab^
TARC	6.9 ± 0.4 ^a^	42.6 ± 1.9 ^c^	20.7 ± 2.2 ^b^	11.2 ± 1.5 ^ab^	42.5 ± 2.5 ^c^	16.2 ± 1.5 ^ab^
IL-6	16.4 ± 2.1 ^ab^	39.0 ± 2.2 ^bc^	36.3 ± 3.1 ^b^	14.5 ± 1.7 ^a^	37.8 ± 2.6 ^bc^	16.6 ± 0.8 ^a^
IL-8	16.5 ± 1.3 ^b^	26.4 ± 0.9 ^c^	40.1 ± 1.5 ^d^	18.5 ± 0.3 ^b^	3.1 ± 1.2 ^a^	25.8 ± 0.7 ^c^
IL-10	5.4 ± 0.6 ^a^	8.9 ± 1.1 ^a^	15.7 ± 4.3 ^bc^	7.3 ± 0.6 ^a^	13.6 ± 1.5 ^b^	15.6 ± 2.3 ^b^
IL-13	16.1 ± 1.4 ^a^	40.4 ± 2.6 ^c^	44.8 ± 2.2 ^c^	26.2 ± 0.5 ^b^	32.6 ± 0.5 ^bc^	28.1 ± 0.9 ^b^

Values represent the means ± S.D. of three determinations. Values with different superscripts in the same columns are significantly different (*p* < 0.05).

**Table 3:** PCR primer used in this experiment.

Gene	Sense	Antisense
MDC	GCATGGCTCGCCTACAGACT	GCAGGGAGGGAGGCAGAGGA
TARC	ATGGCCCCACTGAAGATGCT	TGAACACCAACGGTGGAGGT
IL-6	ATGAACTCCTTCTCCACAAGC	GTTTTCTGCCAGTGCCTCTTTG
IL-8	ATGACTTCCAAGCTGGCCGTGGCT	TCTCAGCCCTCTTCAAAAACTTCT
IL-10	GCCTAACATGCTTCGAGATC	CTCATGGCTTTGTAGATGCC
IL-13	TGAGGAGCTGGTCAACATCA	CAGGTTGATGCTCCATACCAT
MCP-1	ACTGAAGCTCGTACTCTC	CTTGGGTTGTGGAGTGAG
COX-2	CTGGCACCCAGCACAATGAAG	ACCGACTGCTGTCACCTTCA

## Supplementary Materials

The following are available online at https://www.mdpi.com/1420-3049/24/17/3094/s1, Figure S1: ^1^H-NMR spectrum of **1** (300 MHz, Acetone-d_6_); Figure S2: ^1^H-NMR spectrum of **2** (300 MHz, DMSO-d_6_); Figure S3: ^1^H-NMR spectrum of **3** (300 MHz, MeOH-d_4_); Figure S4: ^1^H-NMR spectrum of **4** (300 MHz, MeOH-d_4_); Figure S5: ^13^C-NMR spectrum of **4** (75 MHz, MeOH-d_4_); Figure S6: ^1^H-NMR spectrum of **5** (300 MHz, MeOH-d_4_); Figure S7: ^13^C-NMR spectrum of **5** (75 MHz, MeOH-d_4_); Figure S8: ^1^H-NMR spectrum of **6** (300 MHz, DMSO-d_6_); Figure S9: ^13^C-NMR spectrum of **6** (75 MHz, DMSO-d_6_).

## Abbreviations

QM	*Quercus mongolica* Fisch. ex Ledeb.
PC	Pedunculagin
NF-κB	Nuclear factor-κB
STAT	Signal transducer and activator of transcription
JAK	Janus kinase
COX-2	Cyclooxygenase-2
P38	p38 mitogen-activated protein kinases
IL	Interleukin
MCP-1	Monocyte chemoattractant protein-1
JNK	c-Jun N-terminal kinase
ERK	Extracellular signal-regulated kinase
UVB	Ultraviolet B
TARC	Thymus and activation-regulated chemokine
MDC	Macrophage-derived chemokine
MAPK	Mitogen-activated protein kinase
DMEM	Dulbecco’s modified Eagle medium
HaCaT	Human keratinocytes
RT-PCR	Reverse transcription-polymerase chain reaction
PBS	Phosphate-buffered saline
LPS	Lipopolysaccharides
EtOAc	Ethyl acetate
