# Supplementary material for: Anti-Inflammatory Effects of Phenolic Compounds Isolated from Quercus Mongolica Fisch. ex Ledeb. on UVB-Irradiated Human Skin Cells
Source: Molecules. 2019 Aug 26;24(17):3094. doi: 10.3390/molecules24173094 (PMC6749265; doi:10.3390/molecules24173094)
Supplement: Supplementary file 1 [file molecules-24-03094-s001.pdf]

Supplementary Materials

Anti-inflammatory effects of phenolic compounds isolated from *Quercus mongolica* L. on UVB-irradiated human skin cells

Jun YIN [1,†], Han Hyuk KIM [1,†], In Hyeok HWANG [1], Dong Hee KIM [2] and Min Won LEE [1,*]

[1] Department of Pharmacognosy and Natural product-derived Medicine, College of Pharmacy, Chung-Ang University, Seoul 156-756, Republic of Korea; Jun YIN (yinjun89@naver.com), In Hyeok HWANG (grampus92@naver.com), Han Hyuk KIM (rlagksgur@hanmail.net); Min Won LEE (mwlee@cau.ac.kr)

[2] R&D Department Applied Product Development Team, Traditional Korean Medicine Technology Division, 94, Hwarang-ro(Gapje-dong), Gyeongsan-si, Gyeongsangbuk-do, 38540, Republic of Korea; Dong Hee KIM (kdh83618@naver.com)

* Correspondence: mwlee@cau.ac.kr; Tel.: +82-2-820-5602

S-1. ^{1}H-NMR spectrum of **1** (300 MHz, Acetone-d_6)

S-2. ^{1}H-NMR spectrum of **2** (300 MHz, DMSO-d_6)

S-3. ^{1}H-NMR spectrum of **3** (300 MHz, MeOH-d_4)

S-4. ^{1}H-NMR spectrum of **4** (300 MHz, MeOH-d_4)

S-5. ^{13}C-NMR spectrum of **4** (75 MHz, MeOH-d_4)

S-6. ^{1}H-NMR spectrum of **5** (300 MHz, MeOH-d_4)

S-7. ^{13}C-NMR spectrum of **5** (75 MHz, MeOH-d_4)

S-8. ^{1}H-NMR spectrum of **6** (300 MHz, DMSO-d_6)

S-9. ^{13}C-NMR spectrum of **6** (75 MHz, DMSO-d_6)

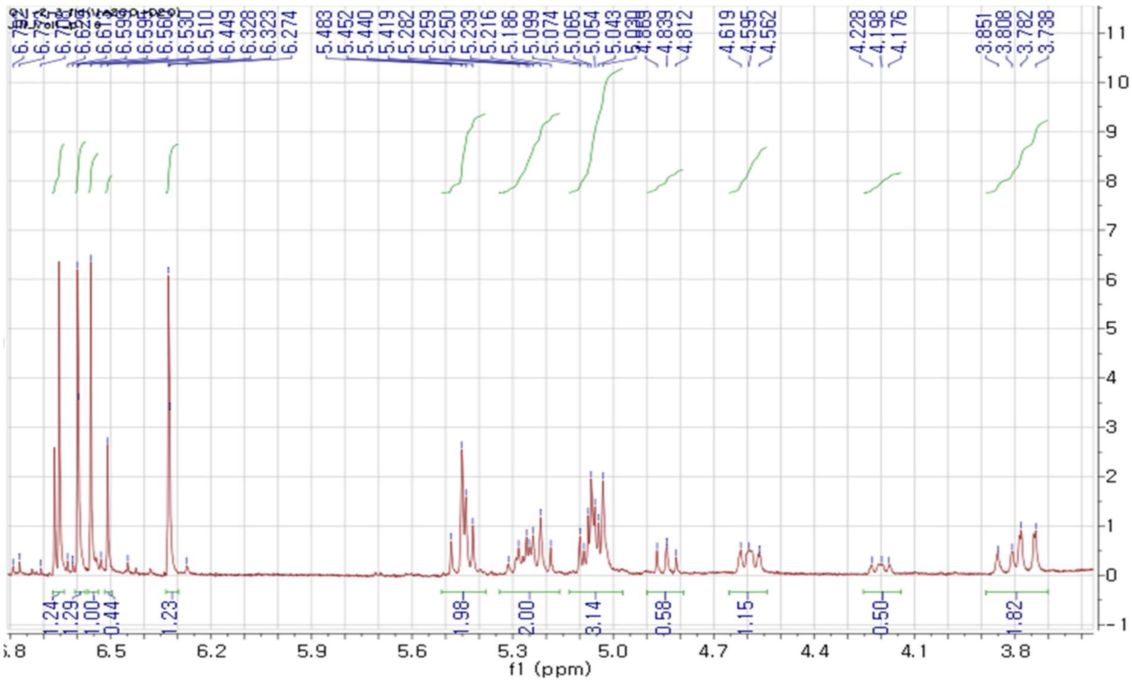

S-1. ^{1}H-NMR spectrum of compound **1** (300 MHz, Acetone-d_6)

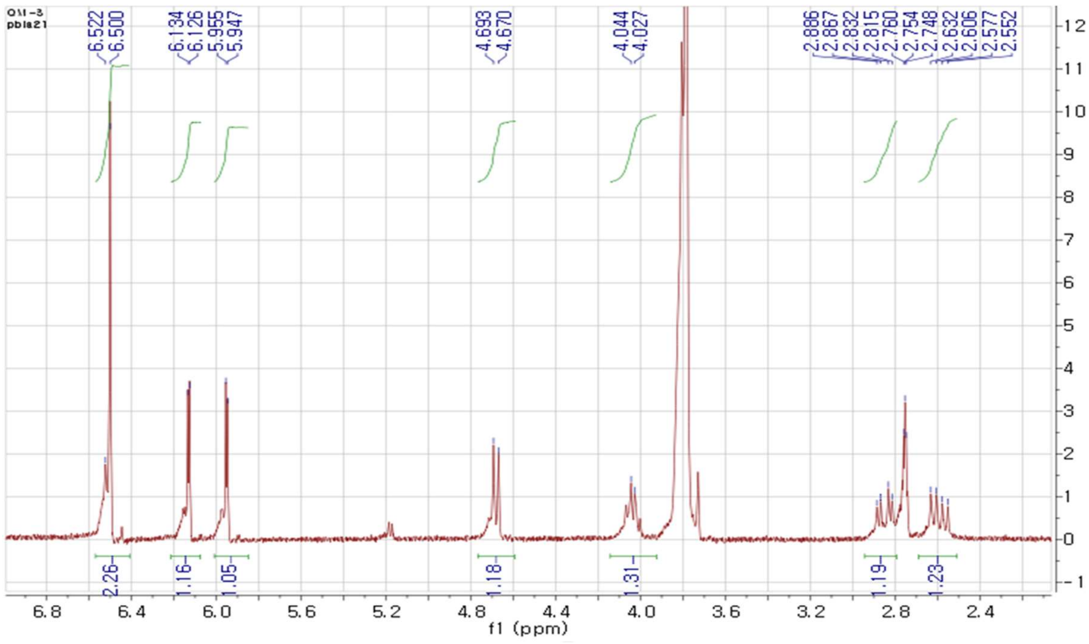

S-2. ^{1}H-NMR spectrum of compound **2** (300 MHz, DMSO-d_6)

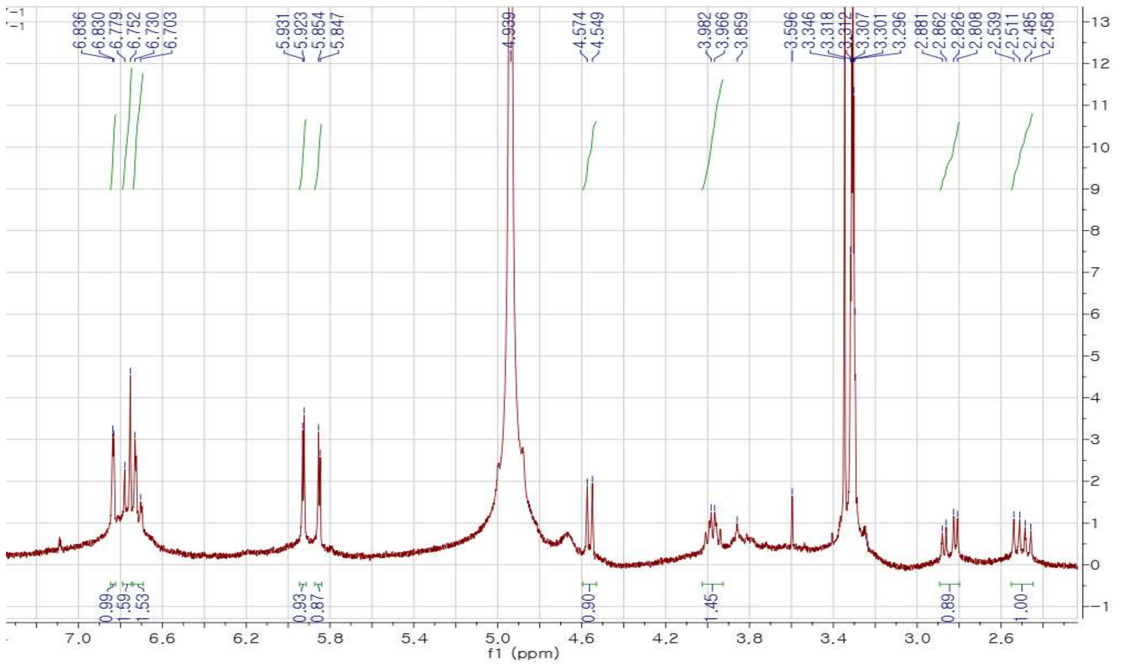

S-3. ^{1}H-NMR spectrum of compound **3** (300 MHz, CD_3OD)

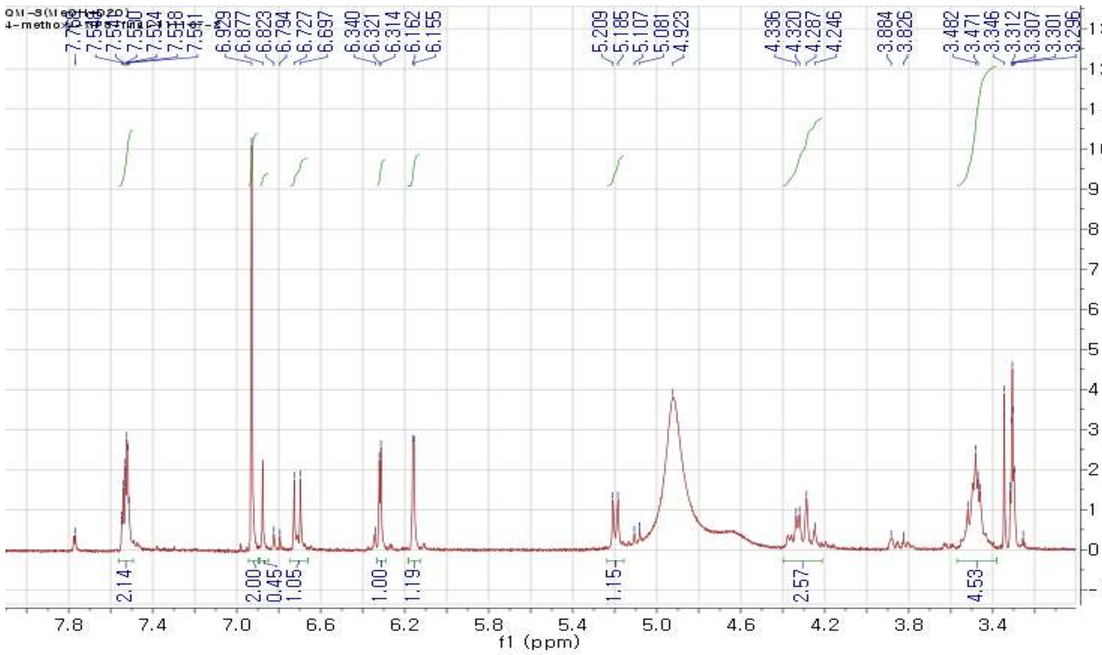

S-4. ^{1}H-NMR spectrum of compound **4** (300 MHz, CD_3OD)

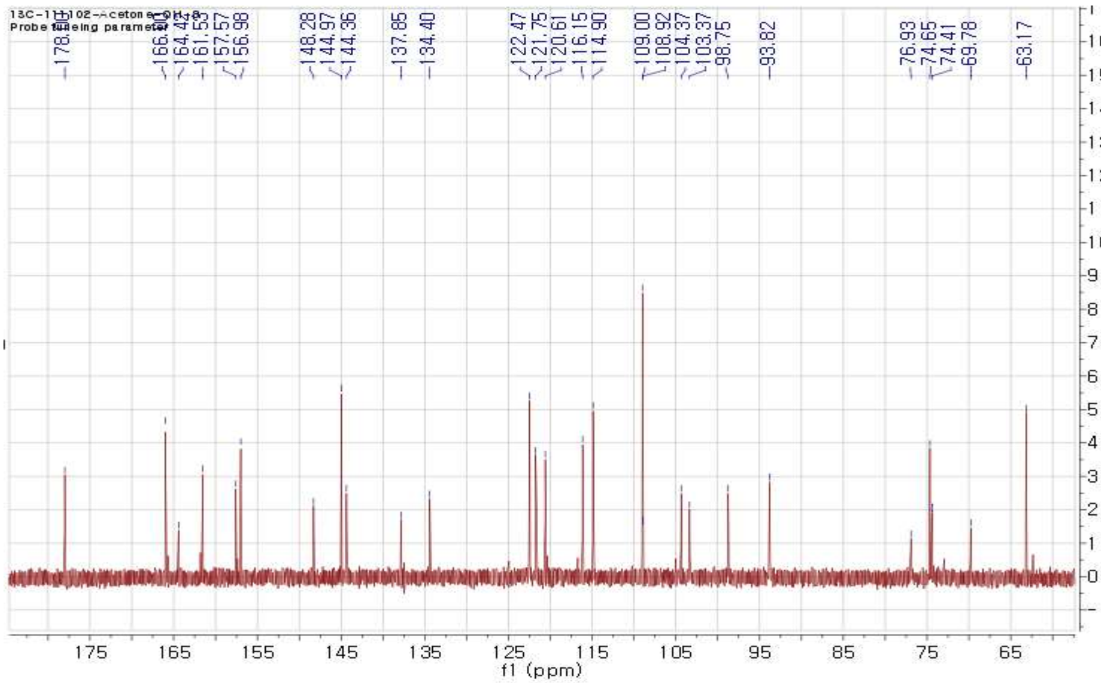

S-5. ^{13}C-NMR spectrum of compound **4** (75 MHz, CD_3OD)

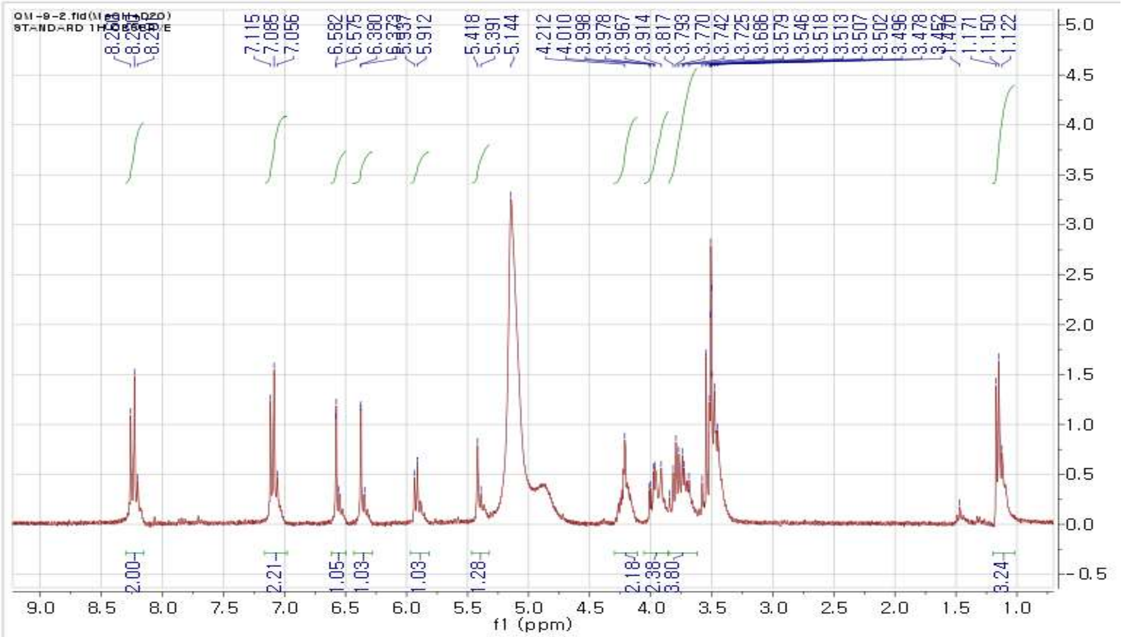

S-6. ^{1}H-NMR spectrum of compound **5** (300 MHz, CD_3OD)

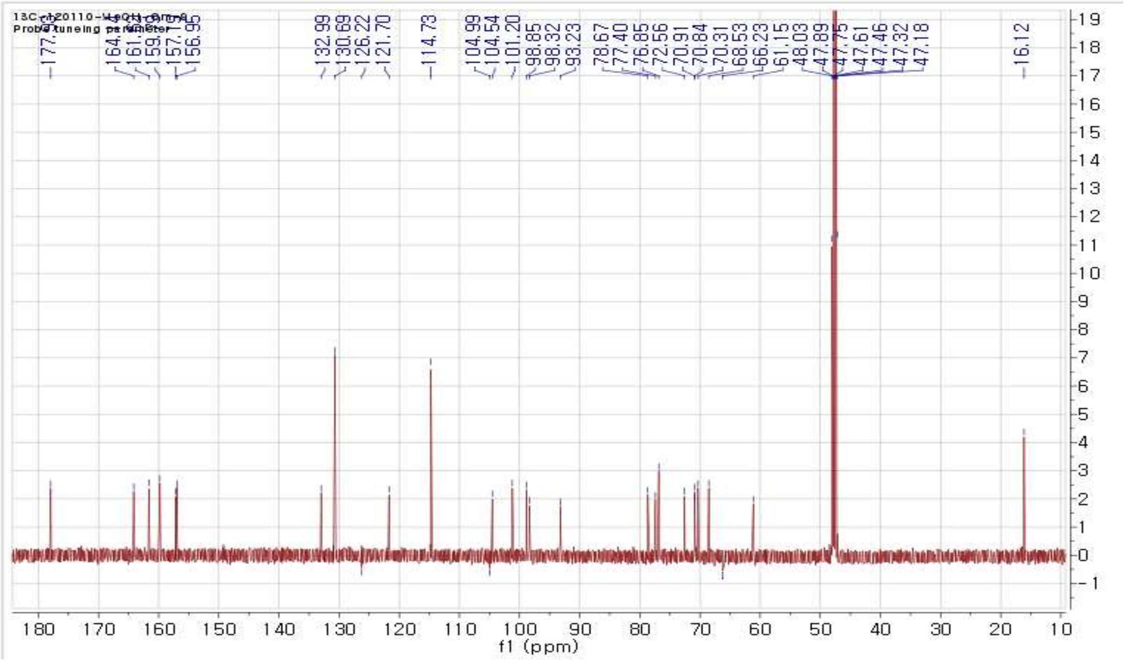

S-7. ^{13}C-NMR spectrum of compound **5** (75 MHz, CD_3OD)

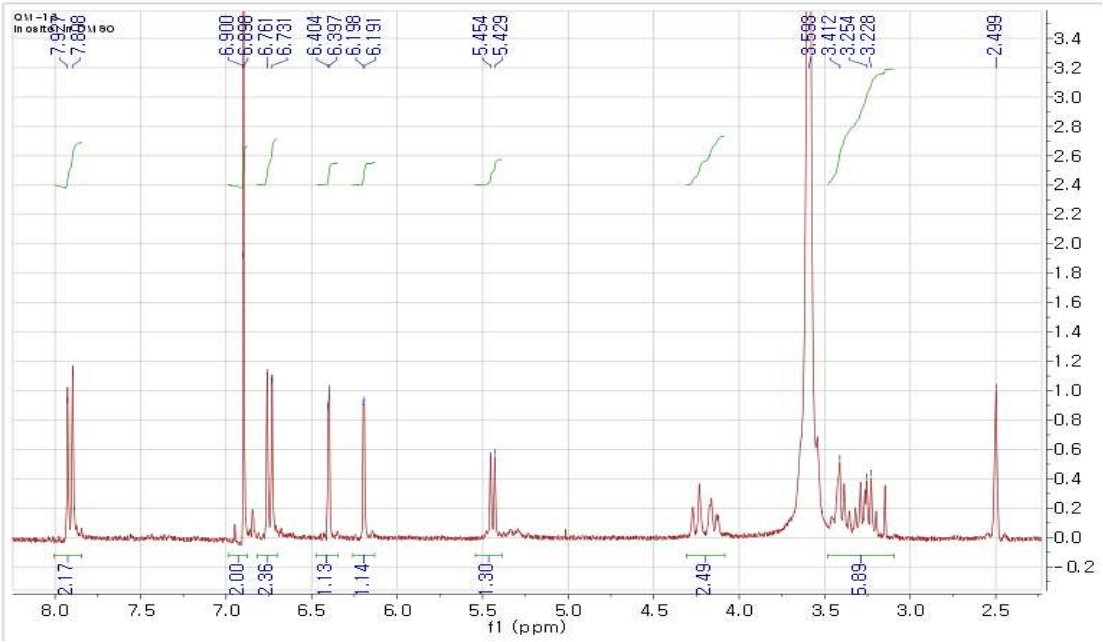

S-8. ^{1}H-NMR spectrum of compound **6** (300 MHz, DMSO-d$_6$)

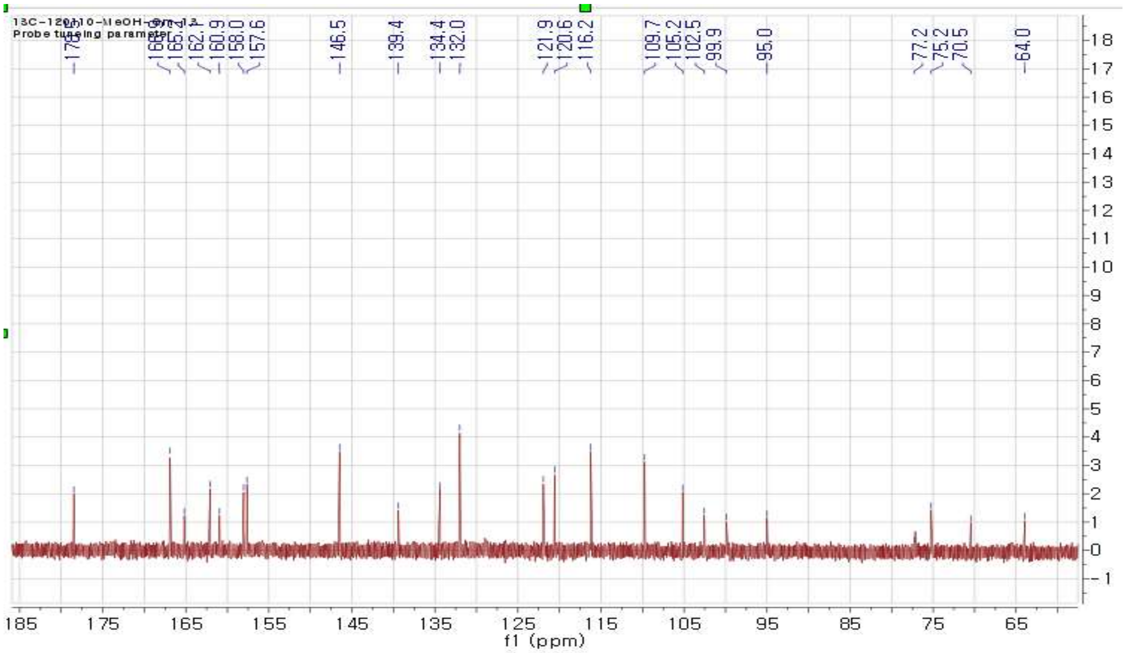

S-9. ^{13}C-NMR spectrum of compound **6** (75 MHz, DMSO-d_6)
